# Global initiatives to prevent climate change

**DOI:** 10.4103/0019-5278.43270

**Published:** 2008-08

**Authors:** Harshal T. Pandve

**Affiliations:** Department of Community Medicine, Dr. D.Y. Patil Medical College, Pimpri, Pune – 411 018, India

Dear Sir,

Climate change is one of the most critical global challenges of our times. Recent events have emphatically demonstrated our growing vulnerability to climate change. Climate change impacts will range from affecting agriculture – further endangering food security – to sea-level rise and the accelerated erosion of coastal zones, increasing intensity of natural disasters, species extinction, and the spread of vector-borne diseases.[1] This issue is of immense importance for every global citizen. That is why it requires an initiative against it globally. Fortunately, a few of the global initiatives to prevent climate change are started by various sectors.

The United Nations Environment Programme (UNEP) stimulates worldwide awareness of the environment and enhances political attention and action through World Environment Day, commemorated each year on 5th June. The World Environment Day slogan for 2008 is “Kick the Habit! Towards a Low Carbon Economy”. Recognizing that climate change is becoming the defining issue of our era, UNEP is asking countries, companies, and communities to focus on greenhouse gas emissions and how to reduce them. The World Environment Day will highlight resources and initiatives that promote low carbon economies and lifestyles, such as improved energy efficiency, alternative energy sources, forest conservation, and eco-friendly consumption.[2] In 2007, The World Environment Day slogan selected was “Melting Ice – a Hot Topic?” In support of International Polar Year, the theme selected for 2007 focuses on the effects that climate change is having on polar ecosystems and communities, and the ensuing consequences around the world.[3] In the past also, UNEP tried to raise voice against climate change. In 1989, the World Environment Day slogan was “Global Warming; Global Warning”; and in 1991, the World Environment Day slogan was “Climate Change. Need for Global Partnership”.[4] World Health Organization (WHO) is also initiating a campaign against climate change. In 2008, World Health Day focuses on the need to protect health from the adverse effects of climate change. The theme “Protecting Health from Climate Change” puts health at the center of the global dialog about climate change. WHO selected this theme in recognition that climate change is posing ever-growing threats to global public health safety. Through increased collaboration, the global community will be better prepared to cope with climate-related health challenges worldwide.[5] Other global initiatives include: on October 29, 2007, leaders of more than 15 governments met in Lisbon, Portugal to launch the establishment of the International Carbon Action Partnership (ICAP). Climate change is a global problem that requires global solutions. ICAP was formed to contribute to the establishment of a well-functioning global cap and trade carbon market. ICAP provides the opportunity for member countries and regions to share best practices and learn from each other's experiences.[6] Since the severe land and forest fires and the transboundary haze pollution incidents of 1997–1998, ASEAN has taken many initiatives and actions on-the-ground to address land and forest fires and the resulting transboundary haze pollution. ASEAN Member Countries, guided by the ASEAN Agreement on Transboundary Haze Pollution, activated bilateral and regional mechanisms to facilitate the timely exchange of information and offer assistance as necessary to deal with the situation expeditiously.[7] Rainforest Action Network (RAN) is made up of 43 staff members in San Francisco, CA and in Tokyo, Japan, plus thousands of volunteer scientists, teachers, parents, students, and other concerned citizens around the world. RAN is working to curb the global warming crisis.[8] The Cooler Heads Coalition was formed on May 6, 1997 under the auspices of the National Consumer Coalition out of concern that the American people were not being informed about the economic impact of proposals to drastically reduce greenhouse gas emissions. Nor was the American public being provided with balanced information about the science of global warming. The Cooler Heads Coalition has a website solely dealing with global warming issues, www.globalwarming.org. This site, designed and maintained by Consumer Alert, pools together information from experts, both inside and outside, of the coalition and serves as a clearinghouse for information on global warming science and policy proposals.[9]

Apart from these initiatives, the prominent personalities and organizations working in the field of climate change are also recognized at international level by the most prestigious organizations and institutes. The Nobel Peace Prize for 2007 was awarded to the Intergovernmental Panel on Climate Change (IPCC) and the former United States Vice President Al Gore for their efforts to build up and disseminate greater knowledge about man-made climate change, and to lay the foundations for the measures that are needed to counteract such change.[10] Another prestigious recognition, the Oscar Award for the Best documentary feature film was given to “An Inconvenient Truth”, a documentary film about global warming, presented by the former United States Vice President Al Gore and directed by Davis Guggenheim.[11] Definitely, such kind of recognition will inspire many workers, especially the youth, to contribute in the initiative to prevent climate change.

To conclude, it is now clear that initiatives to prevent climate change are started, but most importantly these initiatives must be continuous, sustainable, and every individual of all countries will need to contribute to prevent climate change.

## References

[1] About Climate change. http://www.unep.org/themes/climatechange/about/index.asp.

[2] About World Environment Day 2008. http://www.unep.org/wed/2008/english/About_WED_2008/index.asp.

[3] About World Environment Day 2007. http://www.unep.org/wed/2007/english/About_WED_2007/index.asp.

[4] Previous Themes. http://www.unep.org/wed/2008/english/Previous_Themes/index.asp.

[5] World Health Day 2008: Protecting health from climate change. http://www.who.int/world-health-day/en/index.html.

[6] http://www.icapcarbonaction.com.

[7] Actions Taken and Preparations by ASEAN during the Current Dry Season to Mitigate Fires and Address Transboundary Haze Pollution http://www.aseansec.org/18843.htm.

[8] Curb Climate Change http://ran.org/issues/global_warming/.

[9] http://www.globalwarming.org/node/538.

[10] The Nobel Peace Prize 2007 http://nobelprize.org/nobel_prizes/peace/laureates/2007/.

[11] Best Documentary Feature -An Inconvenient Truth. http://www.oscar.com/oscarnight/winners/?pn=detailandnominee=aninconvenienttruthdocumentaryfeaturenominee.

